# *Entamoeba gingivalis* pulmonary abscess - Diagnosed by fine needle aspiration

**DOI:** 10.4103/1742-6413.43179

**Published:** 2008-09-30

**Authors:** Bo Jian, Ana S. Kolansky, Zubair W. Baloach, Prabodh K. Gupta

**Affiliations:** Department of Pathology and Laboratory Medicine, Penn Presbyterian Medical Center, University of Pennsylvania Health System, 39^th^ and Market Streets, Philadelphia, Pennsylvania, 19104, USA; 1 Department of Radiology, Hospital of the University of Pennsylvania, Philadelphia, Pennsylvania, USA; 2 Department of Pathology and Laboratory Medicine, Hospital of the University of Pennsylvania, Philadelphia, Pennsylvania, USA

**Keywords:** *E. gingivalis*, fine needle aspiration

## Abstract

*Entamoeba gingivalis* (*E. gingivalis* ) is a parasitic protozoa of the oral cavity, most often found in gingival tissues around the teeth associated with poor oral hygiene. Here, we report a case of *E. gingivalis* in a pulmonary CT guided fine needle aspiration material, from a 60-year-old man with newly found lung mass. On site Diff-Quik^®^ smear examination revealed the presence of marked acute inflammation, colonies of actinomyces, and a number of ‘large macrophages-like organisms’. Upon examination of the additional material, organisms morphologically consistent with *E. gingivalis* were identified. Pulmonary mass resolved after six weeks of treatment with antibiotics (Clindamycin followed by Penicillin). Proper recognition and distinction between *E. gingivalis* and other species of *Entamoeba* is important for the management of patients.

## Case Report

**Figure 1:**
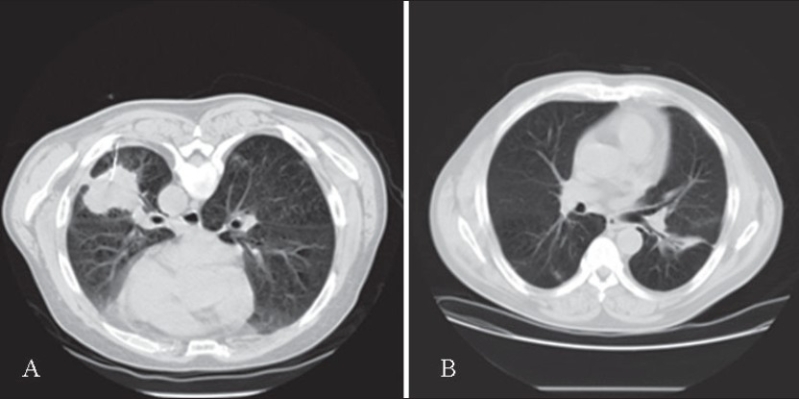
(A) Prone CT chest, placement of 22G Westcott biopsy needle in the left lower lobe mass. (B) Follow up CT chest two months after biopsy. Notice marked improvement with residual inflammation and focal bronchiectasis in the left lower lobe.

A 60-year-old man was admitted for dehydration, weight loss, poor appetite and unstable gait. His past medical history was significant only for alcohol and tobacco use. A CT scan showed a large (50.0 × 50.0 mm) left lower lobe lung mass [Figure 1], during work-up CT-guided fine needle aspiration was performed using #22 gauge Westcott needle. Two passes were made.

**Figure 2:**
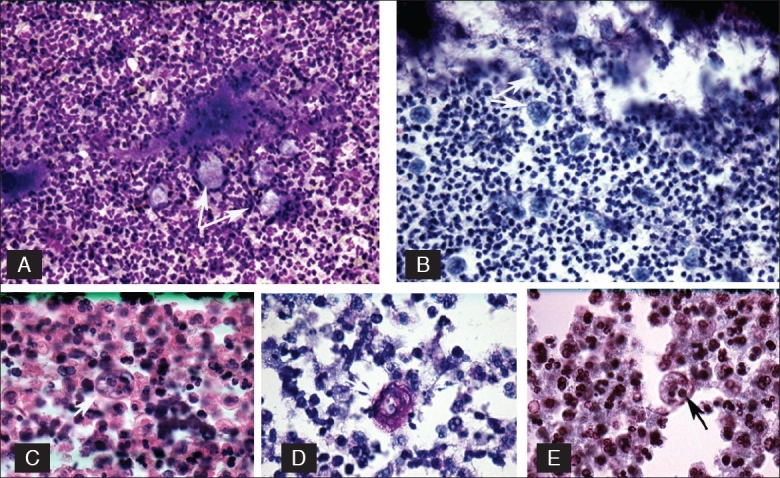
Top row left (A) *E. gingivalis* seen as pale, irregular, macrophage-like structures (arrow), Diff-Quik, (original) ×160. Right (B) *E. gingivalis* (arrow) arranged along the actinomyces. Pale food vacuoles are visible, Pap Stain, (original) ×160. Second row left (C) *E. gingivalis* with thick border, (arrow), H/E. (original) ×400, middle (D) *E. gingivalis*, periodic acid schiff stain (arrow). (Original) ×630, right (E) *E. gingivalis* notice the distinct food vacuoles and karyosome (arrow). Wheatley stain. (Original) ×160.

On-site cytological evaluation revealed marked acute inflammation and colonies of filamentous actinomyces. A few organisms, morphologically resembling large macrophages and embedded within the acute inflammatory exudates, especially along the edge of the filamentous structures, were also identified in the Diff-Quik^®^ stained slides [Figure 2A]. They occurred mostly along the edges of the filamentous actinomyces aggregates [Figure 2B]. The organisms had tinctorial, staining and morphologic features of protozoa, *E gingivalis*. The patient was subsequently discharged, after he showed satisfactory improvement, and treated with antibiotics for six weeks (Clindamycin followed by Penicillin). A chest CT scan examined two months later revealed total resolution of the pulmonary mass, with only a small linear scar left in the left lower lobe. Microbial cultures of the pulmonary aspirate grew mixed Gram positive and negative rods.

## Discussion

The occurrence of *E. gingivalis* in sputum specimens was first reported in Western literature by Sutliff *et al*. in 1951[1]. It is interesting that these authors did not consider it as a causative organism but an oral contaminant, although the dental hygiene condition was not documented in the two reported cases. In addition, *E gingivalis* has been reported in the specimens obtained from the cervix, uterus, and neck lymph node[2–5].

*E. gingivalis* resembles *E. histolytica*-like amoebae and its trophozoite measures 10-35 µm in size. No cysts are observed *in vivo*, although cystic forms have been described in culture studies.[6] Transmission of these parasites is almost entirely (86%), through oral contact.[6] Actinomyces is often found to co-colonize with the *E. gingivalis*. It was first documented in the year 1986, by the Johns Hopkins group, and their precise identification was made in the year 1992, using ribosomal RNA gene sequencing.[7]

Distinction between *E. histolytica* and *E. gingivalis* can be difficult. The morphologic appearances of *E. histolytica* and *E. gingivalis* are quite similar, although the trophozoites of *E. gingivalis* tend to be comparably larger (10-35 µm vs 15-20 µm).

Several important features that may help to distinguish these two parasites include the following:

- *E. gingivalis* has no cyst stage recognizable in the clinical specimens. It has multidirectional pseudopods, best seen in wet mount preparations and Diff-Quik^®^ preparations [Figure 2A].
- *E. gingivalis* is the only species of amoeba that can phagocytose nuclear fragments of white blood cells, ingest bacteria, and an occasional red blood cell. Ingested material is often seen within the large food vacuoles [Figures 2B, C, E].[1,5]
- *E. gingivalis* was seen to have coarser karyosome and a less delicate pattern of peripheral chromatin [Figure 2E], upon examination of Papanicolaou stained smears, as compared to *E. histolytica*. In addition, they are often seen in a markedly inflammed background, with abundant neutrophils. *E. histolytica* ingests erythrocytes, has a fine central karyosome, delicate peripheral chromatin and a single unidirectional pseudopod.

Both organisms also stain with periodic acid schiff reaction, but it is nonspecific [Figure 2D]. Recognition of the protozoa is difficult with the H/E stain [Figure 2C]. These two protozoa can also be distinguished by staining with fluorescein-labeled anti-*E. histolytica* serum. [3] In addition, molecular techniques are highly specific for diagnosis of *E gingivalis*. [4,7] The precise mechanism of pulmonary infection in this case is not known. However, a pulmonary polymicrobial environment, most likely created by the mixed Gram positive and negative rod organisms, contributed to this clinical condition, secondary to aspiration pneumonia. [8]

It is prudent to distinguish between *E. gingivalis* and *E. histolytica*, for the management of patients. Treatment of associated bacteria infection with antibiotic might be enough for *E. gingivalis* infection. However, amebicidal agent (such as Iodoquinol or Paromomycin) or antiprotozoal agent (such as Timidazole) might be necessary to eliminate the *E. histolytica* infection.

## References

[1] Sutliff WD, Green FD, Suter LS (1951). Entamoeba gingivalis in pulmonary suppuration. Am J Trop Med Hyg.

[2] Ruehsen MD, McNeill RE, Frost JK, Gupta PK, Diamond LS, Honigberg BM (1980). Ameba resembling Entamoeba gingivalis in the genital tract of IUD users. Acta Cytol.

[3] Gupta PK (1982). Intrauterine contraceptive device: Vaginal cytology, pathologic changes and their clinical implications. Acta Cytol.

[4] Tsutsumi Y (2000). Entamoeba gingivalis-related endometritis. Case # 145 Atlas of Infectious Disease Pathology (CD ROM).

[5] Perez-Jaffe L, Katz R, Gupta PK (1998). Entamoeba gingivalis identified in a left upper neck nodule by fine-needle aspiration: A case report. Diagn Cytopathol.

[6] Wantland WW, Wantland EM, Winquist DL (1963). Collection, identification, and cultivation of oral protozoa. J Dent Res.

[7] Clark CG, Diamond LS (1992). Colonization of the uterus by the oral protozoan Entamoeba gingivalis. Am J Trop Med Hyg.

[8] Hiroyuki O, Takasha M, Miyuki M, Takayuki N, Mitsuhiro O, Hirotsugu Y (2002). Clinicopathological and cytological study of Entamoeba gingivalis. J Jap Soc Clin Pathol.

